# Static Liquefaction of Tailings Containing Fines: Experimental Exploration, Mechanism Analysis and Evaluation

**DOI:** 10.3390/ma18051123

**Published:** 2025-03-01

**Authors:** Xiaoliang Wang, Hongru Li, Zhenpeng Chen, Yue Zhong, Zaiqiang Hu, Xi Yang, Miaozhi Zhang

**Affiliations:** 1 School of Civil Engineering and Architecture, Xi’an University of Technology, Xi’an 710048, China; 2 Shaanxi Railway Institute, Weinan 714000, China; 3 Shaanxi Key Laboratory of Loess Mechanics and Engineering, Xi’an 710048, China; 4 Ansteel Mining Engineering Corporation, Anshan 114004, China

**Keywords:** fines content, critical state, undrained shear strength, instability line, brittleness index, compressibility, state parameter

## Abstract

Under undrained monotonic static loading, saturated loose granular materials may undergo static liquefaction. Tailings, a kind of granular material, pose particularly serious hazards after static liquefaction. To understand the effects of the initial state and fines content on the static liquefaction of tailings, consolidated undrained triaxial compression tests and one-dimensional compression tests were carried out on tailings with different initial states and fines content. The critical state strength, undrained shear strength, instability line, brittleness index, and compressibility of tailings were investigated, and the tests results were analyzed and discussed using the critical state framework. The results show that tailings with different initial states have the same critical state line, and changes in fines content will cause the position of the critical state line to shift. An increase in the initial void ratio and initial confining pressure will increase the degree of static liquefaction, while the influence of fines content has a threshold value (30%), at which the degree of static liquefaction is the highest. Our analysis shows that compressibility has limitations for evaluating static liquefaction, while the state parameter is an effective indicator for evaluating the static liquefaction of tailings with different initial states and fines contents. The results provide valuable theoretical and practical insights regarding the static liquefaction of tailings and are of great significance for evaluating the stability and preventing the static instability of tailing dams.

## 1. Introduction

**Figure 1:**
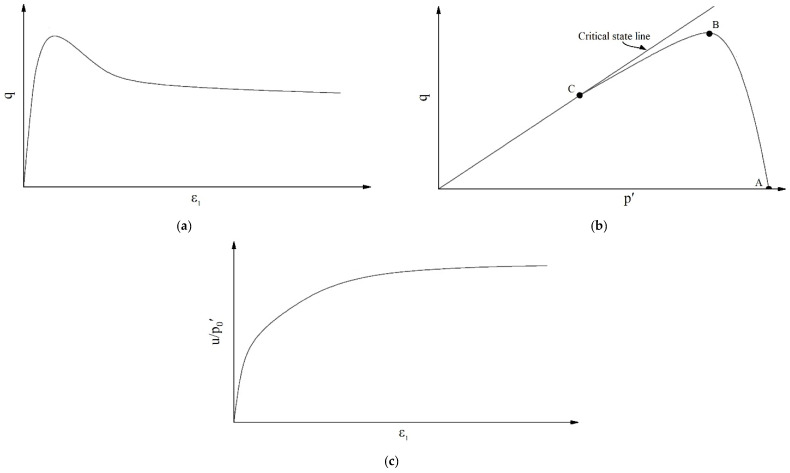
Typical curves in CUTC tests: (**a**) stress–strain curve; (**b**) effective stress path; (**c**) pore water pressure variation curve.

Tailings are the waste material that remains after valuable metals have been extracted from processed rock. As the world’s demand for metallic minerals continues to increase, the quantities of tailings are also constantly increasing. For example, China’s annual tailings emissions are 1.03 billion t. Aside from the 280 million t comprehensively utilized, the rest are all stored in tailings deposits, and there are more than 13,000 existing tailings deposits in China. A tailings dam is a dangerous high-potential-energy source, which not only threatens the safety of downstream residents and facilities but also causes many disasters and environmental problems. Liquefaction is an important cause of tailings dam instability [1,2,3]. Liquefaction of saturated loose granular materials can be initiated by dynamic or static loading under undrained conditions. The behavior of materials under dynamic loading has attracted a lot of interest from researchers and is well understood [4,5,6,7]. However, static liquefaction has been studied to a lesser degree. Static liquefaction [8,9,10,11] refers to the phenomenon in which the shear strength of saturated loose granular materials under undrained static loading decreases rapidly before reaching the damage envelope and is coupled with a rapid increase in pore water pressure. Figure 1 shows typical curves of the static liquefaction of saturated loose tailings in a consolidated undrained triaxial compression (CUTC) test, where u is pore water pressure, ε_1_ is major principal strain, p′ is the mean effective stress, and q is the effective shear stress, and these curves can be expressed using Equations (1) and (2).

(1) $$p^{\prime }=(\sigma_{1}^{\prime }+2\sigma_{3}^{\prime })/3$$

(2) $$q=\sigma_{1}^{\prime }-\sigma_{3}^{\prime }$$

in which σ_1_′ and σ_3_′ are the major and minor principal effective stresses, respectively.

Static liquefaction can cause sudden reductions in strength and accelerated deformation in tailings dams, resulting in catastrophic consequences for personal and property safety [12,13,14,15,16].

In Figure 1b, point A is the starting point of the effective stress path and the start of the test. The stress state at this point can be expressed as (p_0_′, q_0_), where the subscript “0” represents the initial state. Point B is the peak of the effective stress path, at which the shear stress represents the undrained shear strength of the specimen. At point B, the specimen starts to become unstable and soften, and static liquefaction occurs. Therefore, this point is called the instability point. The stress state at this point can be expressed as (p_p_′, q_p_), where the subscript “p” represents the peak state. Point C represents the final state of the shear test, when the specimen is in the ultimate shear failure state with constant effective stress and constant volume, that is, the critical state [17,18]. At this time, the stress state can be expressed as (p_cs_′, q_cs_), where the subscript “cs” represents the critical state, and q_cs_ represents critical state shear strength.

**Figure 2:**
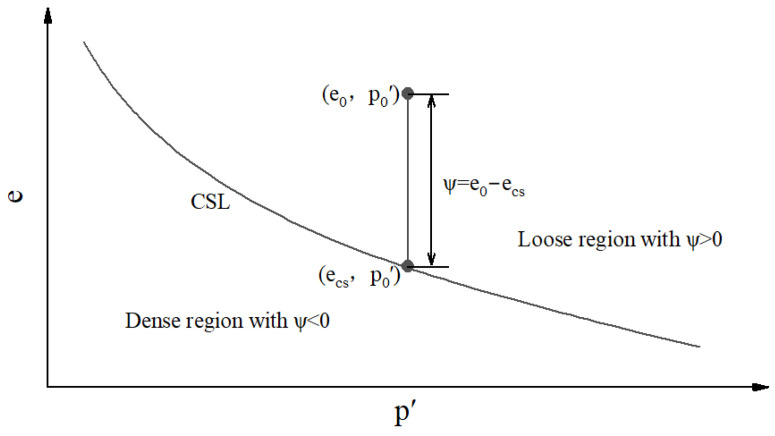
The CSL and definition of ψ.

Numerous studies [19,20,21] have been performed to research static liquefaction characteristics, especially for sands subjected to undrained loading [22,23,24]. These studies have mainly focused on the effect of the initial state (the void ratio e_0_ and the mean principal stress p_0_′ after consolidation) at point A on the location of points B and C. For a given type of sand, a unique critical state line (CSL) is obtained in the plane of the void ratio versus the mean principal stress, as shown in Figure 2 [25,26]. The void ratio remains constant in the CUTC tests, and the critical state is related only to e_0_ and not to p_0_′. The critical state stress ratio M, calculated using Equation (3), is constant, independent of the initial state [27].

(3) $$M=\frac{q_{cs}}{p_{cs}^{\prime }}$$

In CUTC tests, the collapse line that connects points B and C determined using specimens with the same e_0_ but different p_0_′ was proposed [28]. In the p-q plane, the position of the collapse line moves down along the CSL, but the slope remains unchanged as the e_0_ increases. Similar results were obtained by Ishihara [25,29]. After further investigation, Lade found that the collapse line is a straight line connecting point B to the origin and renamed it the instability line (IL) [30,31]. The slope of the IL can be calculated using Equation (4).

(4) $$\eta_{IL}=\frac{q_{p}}{p_{p}^{\prime }}$$

η_IL_ increases with a decreasing e_0_, as verified by [32,33]. Recently, the research by J. Yang showed that η_IL_ is not only related to e_0_ but also decreases with the increase in p_0_′ [34,35].

Tailings, as well as natural sands, often contain fines (with a particle size less than 0.075 mm), and the fines content (FC) can change the position of the CSL in e-p plane [36,37,38,39]. There has been no unified conclusion on the influence of the existence of fines on static liquefaction. Several studies have concluded that the existence of fines decreases the liquefaction potential [40,41,42], while an opposite conclusion has been made by [43,44]. Some studies have found that there exists a threshold FC [45,46,47]. When the FC is below the threshold content, the fine grains will slip into the spaces of the large-grained skeleton as shear stress is applied. This phenomenon increases the contraction of the specimen, with a consequent increase in pore water pressure and liquefaction potential. On the contrary, when the FC exceeds the threshold content, the fine grains will fill the spaces of the skeleton, halting the specimen’s contraction or even making it expand, and the liquefaction phenomenon ceases. In order to evaluate the contraction of specimens, Yamamuro and Lade proposed the concept of compressibility based on the results of one-dimensional compression (OC) tests conducted on sandy soils [48,49] and investigated the relationship between compressibility and static liquefaction. The results showed that compressibility is a direct indication of the liquefaction potential and related to the development of pore water pressure. On the other hand, J. Yang [35] studied the effects of initial shear stress and the initial state on slope stability and liquefaction in granular soils using the critical state framework and concluded that the IL is only correlated with the initial state parameter. The state parameter (ψ) [19] is illustrated in Figure 2, and it can be specifically calculated using Equation (5).

(5) $$\psi =e_{0}-e_{cs}$$

where e_cs_ is the void ratio at the same mean effective stress as e_0_ on the CSL.

In summary, in the existing literature, in-depth research has been conducted on the static liquefaction problems of coarse-grained soils, but there the following issues still need to be addressed:

(i) The impact of fines on the static liquefaction of coarse-grained soil is still unclear, and there is a lack of experimental data on the effect of FC;
(ii) The compressibility evaluation parameters of soil are not uniform, and there is a lack of systematic research on their relationship with static liquefaction;
(iii) There is still a lack of experimental research on the static liquefaction problems pertaining to tailings, a special granular material.

In response to the above issues, we selected gold tailings as experimental materials and studied the static liquefaction behavior of tailings with different fines contents and initial states through CUTC tests and OC tests under the critical state framework. The specific objectives were as follows:

(i) To study the effects of the initial state and FC of tailings on the critical state, the instability line, undrained shear strength, and the brittleness index;
(ii) To compare two existing compressibility parameters and analyze their advantages and disadvantages;
(iii) To identify the relationship between tailings’ compressibility and static liquefaction and evaluate static liquefaction using compressibility parameters within the critical state framework.

The results of this study have guiding significance for the design and monitoring of ability to warn of risks to tailings dams, providing an experimental and theoretical basis for the stability assessment of existing tailings dams.

The structure of this paper is as follows. Section 2 introduces the experimental materials and methods. Section 3 analyzes the results of OC tests and CUTC tests. Section 4 discusses the mechanism of the static liquefaction of tailings with fines and the role of compressibility indicators in evaluating static liquefaction, and it provides suggestions for future research. Section 5 summarizes the main findings of this paper.

## 2. Materials and Methods

### 2.1. Materials

Gold tailings were used as test materials in this study. They were taken from the upstream dry beach of a gold tailings deposit in Ili, Xinjiang, China. In order to ensure the representativeness of the samples taken, five sampling points were set up along the direction perpendicular to the dam axis on the beach surface. The sampling depth was about 10 cm below the beach surface, and the tailings were loose in texture and yellowish-green in color. The basic physical property indexes are shown in Table 1.

**Figure 3:**
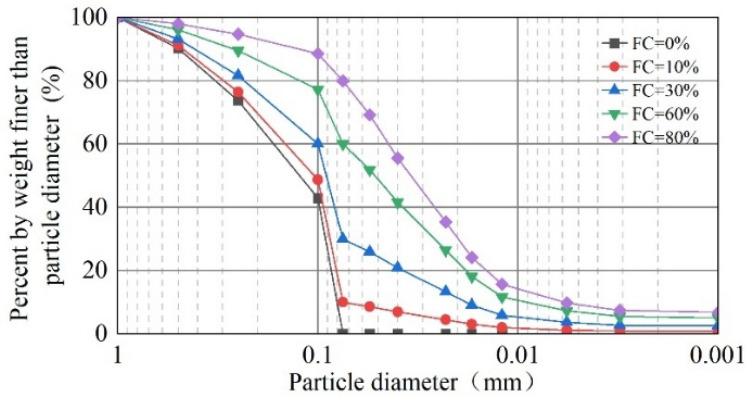
Particle-size distribution of tailings with different FC.

The FC of the tailings was between 72.53% and 81.05%. It is evident that FC in tailings varies with location, with a maximum of over 80%. In order to study the influence of FC on the static liquefaction characteristics of tailings, excluding the influence of the coarse particle gradings of different sampling points, the sieved-out fine particles of the P5 tailings were taken as the base tailings. The base tailings were mixed with tailings’ fines and formulated into mixed-tailings material with FC of 0%, 10%, 30%, 60%, and 80% according to the mass ratio. The particle-grading curves of tailings with different FC are plotted in Figure 3.

**Figure 4:**
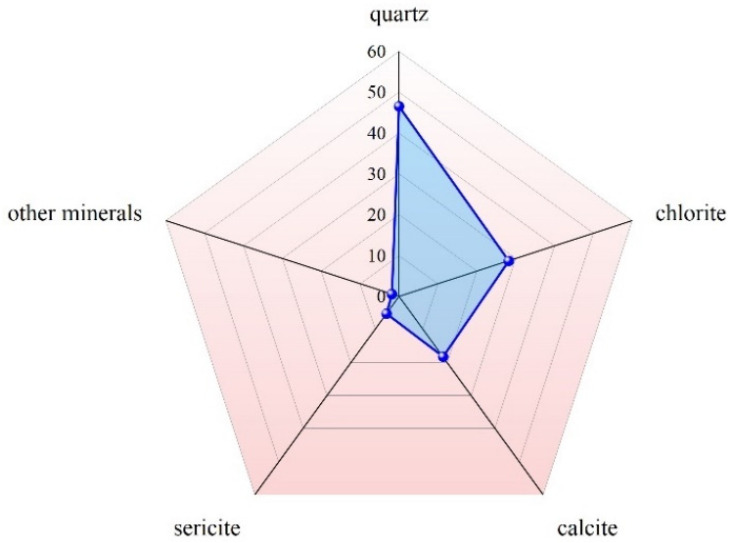
Mineral composition of the tailings.

The mineral composition was measured using an electron microprobe, as shown in Figure 4. The principal mineral constituents of these metal tailings are quartz, chlorite, calcite, and sericite.

### 2.2. Methods

#### 2.2.1. CUTC Tests

CUTC tests were conducted to study the static liquefaction characteristics of tailings under different initial states and FCs using a SYL-2 stress path triaxial apparatus (Nanjing soil instrument factory, Nanjing, Jiangsu, China). The CUTC tests for gold tailings and instrument calibration were performed in accordance with the “Test Methods of Soils for Highway Engineering” (JTG 3430-2020) [50]. The stress path triaxial apparatus includes a pressure supply system, a control system, and a data acquisition system. The control system consists of a strain control program and a stress control program, which can carry out conventional triaxial tests and stress path triaxial tests. The strain control program is used in this study.

The moist tamping method [25] was used for specimen preparation. The specimens were cylinders with a diameter of 39.1 mm and a height of 80 mm. The required mass of each specimen was determined based on the predetermined FC and void ratio. Five equal pre-weighed portions of tailings with water content of 5% were scattered sequentially into the sample making mold and compacted in 5 layers for one specimen. Then, the prepared specimen was sleeved inside the latex membrane and mounted on the triaxial pressure chamber base. The specimens were saturated using distilled water with a back pressure of 300 kPa. The criterion for saturation of each specimen was that the B-value had to be more than 0.98. The test FCs were 0%, 10%, 30%, 60%, and 80%, and the consolidation mean effective stress values were 50 kPa, 100 kPa, 200 kPa, and 300 kPa. The process of shearing was carried out while the specimens were still undrained, with a loading rate of 0.08 mm/min. The tests were finished when the axial strain reached 25%. Table 2 shows the specific test program.

#### 2.2.2. OC Tests

To describe volumetric compressibility and its dependence on FC, void ratio, and pressure, a series of OC tests were performed on the tailings. The instrument used was an OC test apparatus (Nanjing soil instrument factory, Nanjing, Jiangsu, China). The OC tests for gold tailings and instrument calibration were performed in accordance with the “Test Methods of Soils for Highway Engineering” (JTG 3430-2020) [50]. The sample preparation method was the same as that used for the CUTC tests, and the specimen’s size was 61.8 mm in diameter and 40 mm in height. The vertical pressure (σ_v_′) of the tests was loaded from 0 kPa to 800 kPa. The void ratio was calculated using vertical displacement. Table 3 shows the specific test program.

## 3. Results and Analysis

### 3.1. Analysis of CUTC Tests

**Figure 5:**
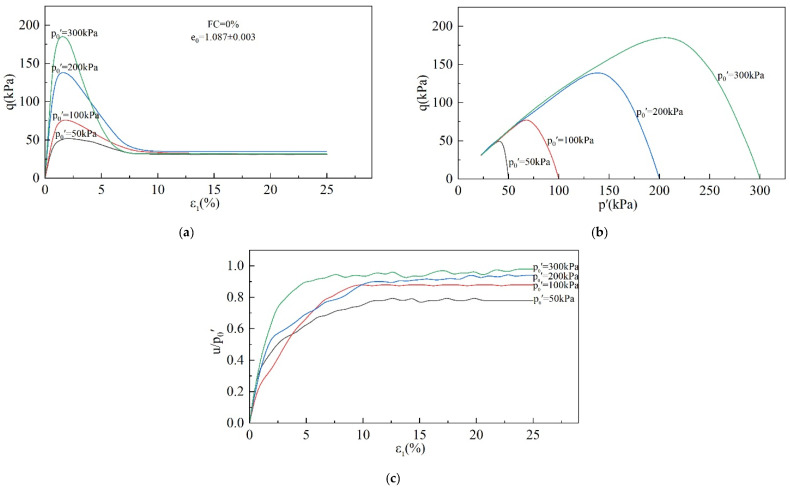
Effect of p_0_′ on undrained behavior of tailings: (**a**) stress–strain diagram; (**b**) effective stress path diagram; (**c**) pore water pressure ratio diagram.

**Figure 6:**
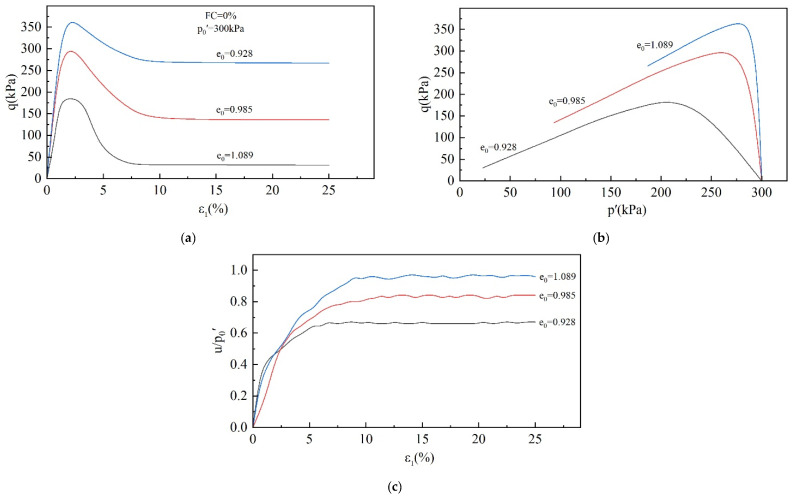
Effect of e_0_ on undrained behavior of tailings: (**a**) stress–strain diagram; (**b**) effective stress path diagram; (**c**) pore water pressure ratio diagram.

**Figure 7:**
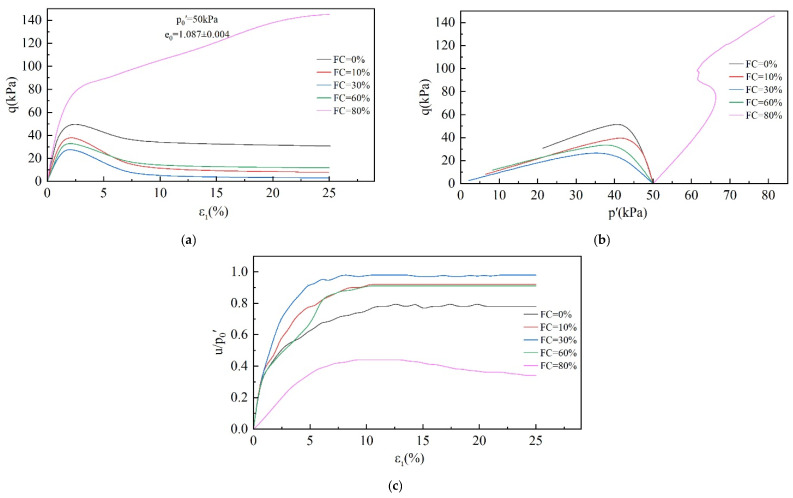
Effect of FC on undrained behavior of tailings: (**a**) stress–strain diagram; (**b**) effective stress path diagram; (**c**) pore water pressure ratio diagram.

The stress–strain curves of the specimens were obtained using CUTC tests, and the corresponding effective stress path curves and pore water pressure ratio curves were also plotted, as shown in Figure 5, Figure 6 and Figure 7.

Figure 5 shows the effect of the p_0_′ on the undrained behavior of the tailings. Static liquefaction occurred in all four specimens. As shown in Figure 5a, as the strain develops, the shear stress increases and reaches a peak at about ε_1_ = 5% before softening. The shear stress decreases sharply and tends to a stable value after ε_1_ = 10%. q_p_ increases with an increasing p_0_′. Figure 5b,c show that the mean effective stress decreases as shear stress is applied, which is mainly due to the increasing pore water pressure. The pore water pressure ratio indicates the degree of development of pore pressure, which increases with an increasing p_0_′.

Figure 6 shows the effect of the e_0_ on the undrained behavior of the tailings. Similar to the effect of p_0_′, the void ratio mainly affects the peak shear stress and pore water pressure. As e_0_ grows, the tailings become loose, peak shear stress decreases, and pore water pressure increases.

As can be seen in Figure 7, static liquefaction occurred in all the specimens with an FC in the range of 0–60%, and the shear stress reached its peak before the strain reached 5%, followed by softening, and tended to a stable value after the strain reached 10%. The pore water pressure, on the other hand, continued to increase and achieved stability when ε_1_ reached 10%. However, the final pore water pressure ratio shows a tendency of increasing and then decreasing with the increase in FC and reached a maximum value at FC = 30%. The specimen with an FC = 80% did not exhibit a static liquefaction phenomenon, and the shear stress continued to increase during the shear process. When the strain reached 25%, the shear stress had not yet reached stability. On the other hand, the pore water pressure ratio increased slowly in the early stage, achieved a brief period of stability at about 10% axial strain, and then showed a decreasing trend, proving that shear dilation occurred. From the above analysis, it can be found that the undrained behavior of tailings has a significant dependence on p_0_′, e_0_, and FC.

The behavior of specimens undergoing static liquefaction can be summarized in three stages. The first is (i) the stage of increasing shear stress. In this stage, as the shear strain increases, the pore water pressure and the shear stress increase, eventually reaching the peak strength. (ii) The next is the strength-softening stage. (iii) Subsequently, there is the strength stability stage. As the shear strain continues to develop, the shear strength remains unchanged, and the pore water pressure reaches its maximum value and remains unchanged. The division point between stages (i) and (ii) represents the undrained shear strength of the specimen and the onset of static liquefaction or instability. In the next stage (iii), the specimen reaches the critical state. Obviously, the instability point and the critical state point are critical for studying static liquefaction. These two points mark the beginning and the final state of static liquefaction and determine the severity of static liquefaction.

#### 3.1.1. Critical State

**Figure 8:**
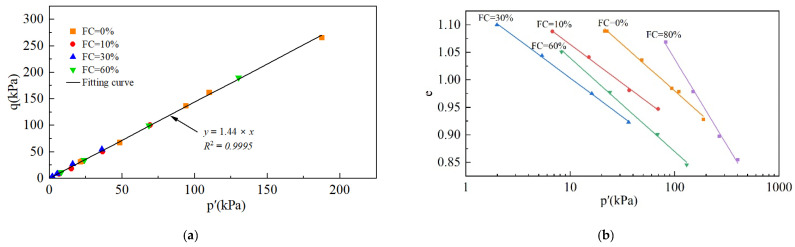
The CSLs: (**a**) the CSL in q-p′ space; (**b**) the CSLs in e-p′ space.

In this study, the state at ε_1_ = 25% was taken as the critical state. The CSLs were fitted from critical state points and are shown in Figure 8.

With the change in FC, the critical stress ratio M becomes almost constant, with a mean value of M = 1.44. It can be assumed that FC has no effect on M, as described in the results reported by Bouckovalas [27]. Previous studies [38,51] have shown that the CSL can be expressed as a straight line on the e-lnp′ plane using Equation (6):

(6) $$e=\Gamma -\lambda lnp^{\prime }$$

where Γ is the intercept of the vertical coordinate at p′ = 1 kPa, and λ represents the slope of the CSL in e-lnp space. Figure 8b shows that the CSLs are approximately parallel to each other as FC increases in the range of 0–60%, with λ being a constant regardless of the FC. This is because λ increases with increasing particle angularity [36,38]. The fines and base tailings used in the tests were taken from the same tailings and had the same particle angles. However, the variation in Γ was due to the change in FC with different particle size distributions [38]. The CSLs at FC = 80% are not parallel to the other CSLs, mainly because the specimens with an FC = 80% exhibit shear dilation and do not reach the critical state at ε_1_ = 25%. However, a trend in Γ relating to differences in FC could be determined; that is, Γ decreased and then increased as the FC increased from 0% to 80%, with a minimum value at FC = 30%.

#### 3.1.2. IL

**Figure 9:**
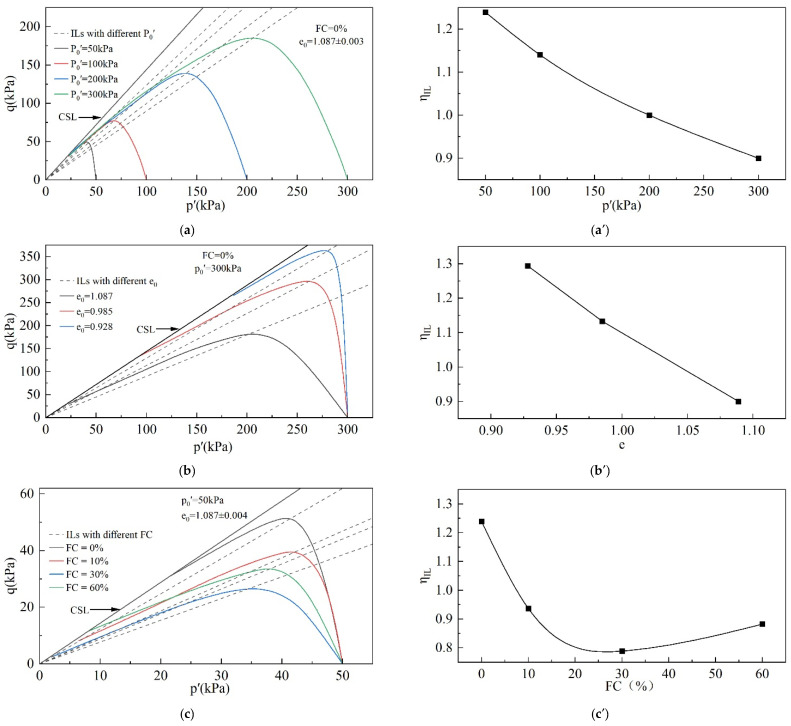
Tailings’ ILs: (**a**,**a′**) effect of p_0_′; (**b**,**b′**) effect of e_0_; (**c**,**c′**) effect of FC.

It is interesting to examine how the IL is affected by the initial state and FC of tailings. Figure 9 illustrates the ILs for different initial states and FCs.

In Figure 9, η_IL_ is closely related to p_0_′, e_0_, and FC. η_IL_ decreases monotonically as p_0_′ and e_0_ increase. This result indicates that static liquefaction is more likely to be triggered at larger e_0_ and higher p_0_′. When the e_0_ and p_0_′ are constant, η_IL_ decreases and then increases with the increase in FC. η_IL_ reaches its lowest value at FC = 30%. This indicates that the change in FC cannot be simply interpreted as beneficial or detrimental to the stability of tailings. For the tailings used in this study, when the FC is less than 30%, the increase in the FC is detrimental to the tailings’ stability, while when the FC is greater than 30%, the increase in FC helps to improve the tailings’ stability and reduce the possibility of static liquefaction. When the FC = 30%, it is most detrimental for the stability of tailings.

#### 3.1.3. Undrained Shear Strength

In this study, for specimens undergoing static liquefaction, the undrained shear strength is equivalent to q_p_, which represents the maximum shear stress for resisting static liquefaction.

**Figure 10:**
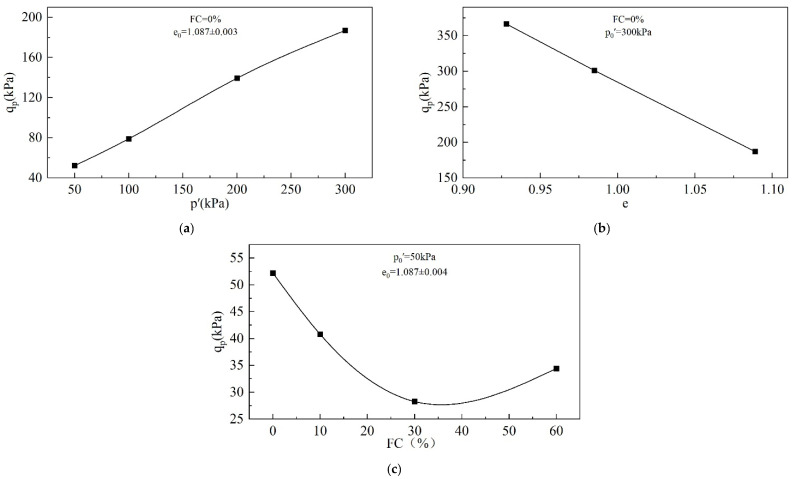
Undrained shear strength of tailings: (**a**) effect of p_0_′; (**b**) effect of e_0_; (**c**) effect of FC.

Figure 10 presents the variation in q_p_ with p_0_′, e_0_, and FC. q_p_ increases with an increasing p_0_′, rising from 52.2 kPa at p_0_′ = 50 kPa to 187 kPa at p_0_′ = 300 kPa. q_p_ decreases with an increasing e_0_, falling from 366.4 kPa at e_0_ = 0.928 to 187 kPa at e_0_ = 1.089. The increase in e_0_ leads to a growth in pore water pressure (Figure 6c), which in turn leads to a reduction in mean effective stress and ultimately a reduction in undrained shear strength. The effect of FC on q_p_ is not monotonic, but there is a threshold. When the FC is below the threshold, q_p_ decreases with an increasing FC, but the trend is opposite when the FC exceeds the threshold. The FC threshold for the tailings is around 30%.

#### 3.1.4. Brittleness Index

The brittleness index (I_B_) is commonly employed to evaluate the extent of strength softening or the severity of liquefaction.

(7) $$I_{B}=\frac{q_{p}-q_{cs}}{q_{p}}$$

I_B_ ranges from 0 to 1. An undrained I_B_ = 1 indicates that the q_cs_ of the tailings specimen is 0 kPa, and complete static liquefaction occurs. When the undrained I_B_ = 0, this indicates that the q_cs_ of the tailings is the same as the q_p_, that is, the q-ε_1_ curve is strain-hardening, and the tailings do not have the potential for static liquefaction.

**Figure 11:**
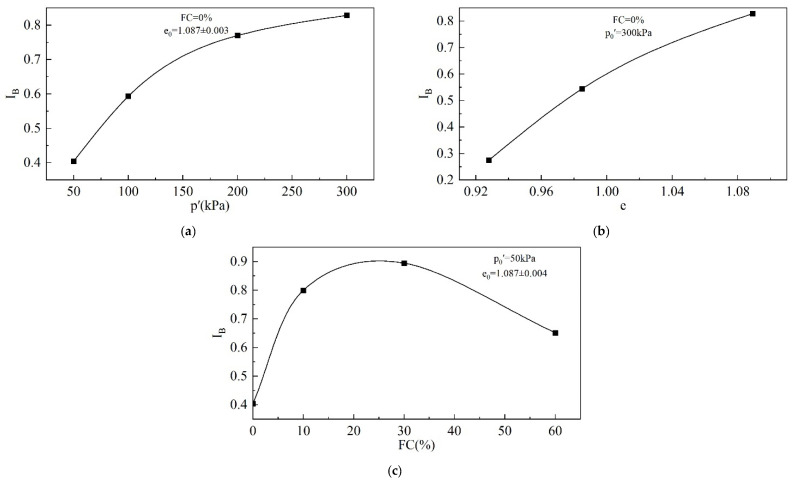
I_B_ of tailings: (**a**) effect of p_0_′; (**b**) effect of e_0_; (**c**) effect of FC.

Figure 11 presents the variation in I_B_ with e_0_, p_0_′, and FC. I_B_ increases with the increase in p_0_′, but the trend slows down gradually. When p_0_′ increases from 50 kPa to 300 kPa, the I_B_ increases from 0.404 to 0.828, a more than twofold increase. I_B_ increases with the increase in e_0_, indicating a growth in the softening degree. When e_0_ = 1.089, I_B_ reaches as high as 0.828, which is close to the point of complete liquefaction. Obviously, the increase in both the p_0_′ and e_0_ will increase the risk of the tailings’ static liquefaction and the severity of this risk. Figure 11c shows that there exists a threshold for the effect of FC on I_B_. As the FC increases, I_B_ increases first and then decreases. The FC threshold for the tailings is around 30%.

### 3.2. Analysis of OC Tests

**Figure 12:**
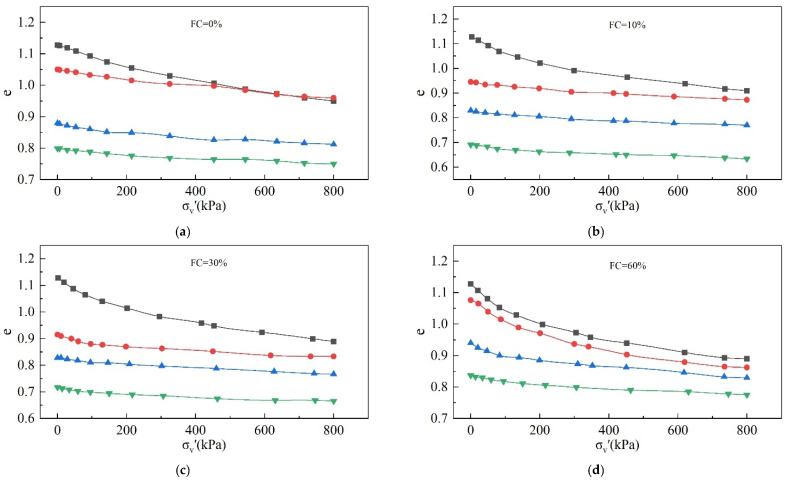

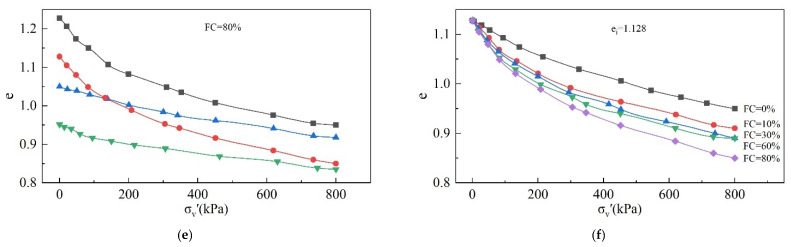
Compression curves of tailings with different ei and FCs: (**a**) FC = 0%; (**b**) FC = 10%; (**c**) FC = 30%; (**d**) FC = 60%; (**e**) FC = 80%; (**f**) ei = 1.128.

The OC tests results are shown in the Figure 12.

As shown in Figure 12, with the increase in σ_v_′, the void ratio and the slope of the compression curves gradually decrease. The compression curves exhibit different compressibility with different initial void ratios and FCs.

Compressibility [48,49] can be defined as the slope of the compression curve at different points:

(8) $$m=\frac{de}{d\sigma_{v}\prime }$$

As can be seen from the Equation (8), compressibility represents the variation in the void ratio caused by the variation in vertical pressure per unit. The greater the compressibility, the easier it is for a material to be compressed.

**Figure 13:**
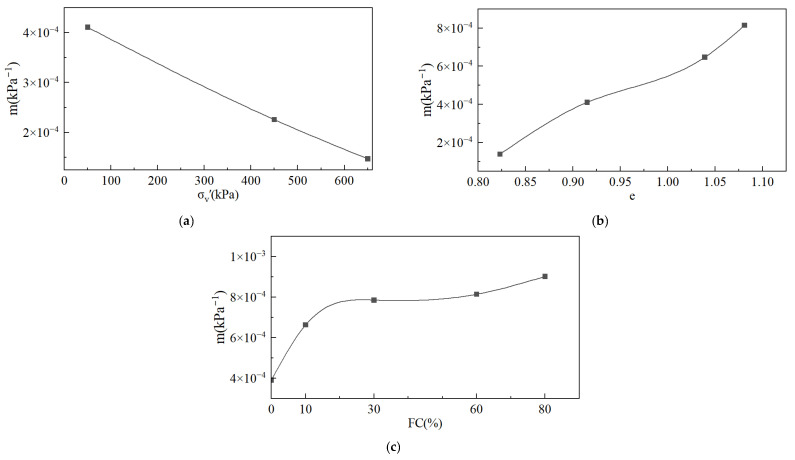
Compressibility of tailings: (**a**) effect of σ_v_′; (**b**) effect of e_0_; (**c**) effect of FC.

Taking an FC = 60% as an example, we analyzed the changes in compressibility for the same FC but different e_i_ and σ_v_′. Figure 13a shows the change rule for compressibility with respect to σ_v_′ when e_i_ = 0.9. With the increase in σ_v_′, the compressibility of the tailings decreases. The change rule for compressibility with respect to the void ratio when σ_v_′ = 50 kPa is shown in Figure 13b. Under the same σ_v_′, the compressibility of the tailings increases with the increase in the void ratio.

For simplicity, the variation in compressibility with respect to FC can be expressed by the slope of the cut line from 0 kPa to 50 kPa; that is, m = de/dσ_v_′ ≈ Δe/Δσ_v_′ = Δe/50 kPa. The compression curves of different FCs are shown in Figure 13c when the initial void ratio e_i_ = 1.128. It is obvious that compressibility increases with an increasing FC.

## 4. Discussion

### 4.1. Relationship Between Compressibility and Static Liquefaction

**Figure 14:**
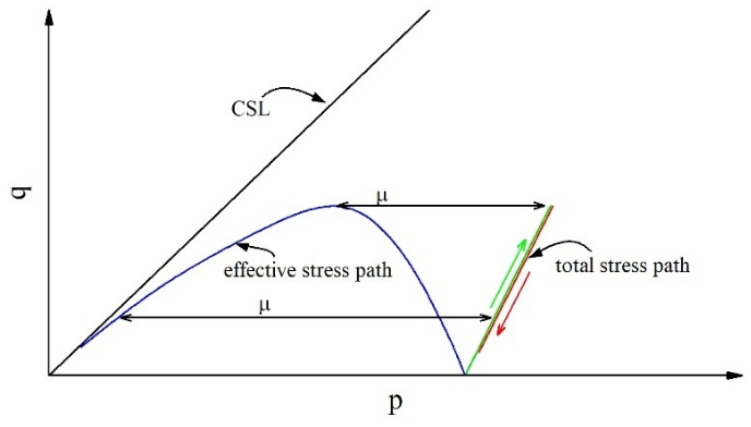
Typical stress path diagram of CUTC tests.

Figure 14 shows a typical stress path diagram for static liquefaction tailings under CUTC test conditions-. In the effective stress path, during the whole shear process, the stress ratio of the tailings and the effective internal friction angle continuously increase. Therefore, the ability of the tailings to resist shear will continue to increase if the mean effective stress is unchanged. The reason why the shear strength starts to decrease and static liquefaction occurs after q_p_ is not the decrease in the internal friction angle but rather the decrease in mean effective stress caused by the increase in pore water pressure.

Equation (9) is satisfied by the CUTC tests, and Equation (10) is the differential form. From Equation (10), it can be gleaned that although the stress ratio continues to increase (dη > 0), if the increment in pore water pressure exceeds that for total mean effective stress, the shear stress may decrease. The faster the growth in pore water pressure, the earlier the peak shear stress is reached, and the lower the corresponding η_IL_.

(9) $$\left\{\begin{array}{c} q=\eta p^{\prime }\\ p^{\prime }=p-u \end{array}\right.$$

(10) $$\left\{\begin{array}{c} \mathrm{dq}=d\eta p^{\prime }+\eta {dp}^{\prime }\\ {dp}^{\prime }=\mathrm{dp}-\mathrm{du} \end{array}\right.$$

**Figure 15:**
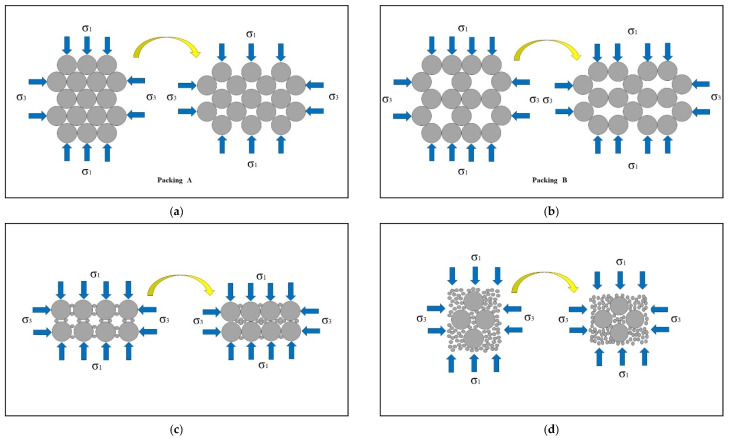
Diagrams of particles’ behavior under compression: (**a**) Packing A; (**b**) Packing B; (**c**) tailings with a low FC; (**d**) the fines completely fill the void.

The development of pore water pressure in soil during the shearing process is related to the density and particle arrangement of the soil [52]. The particle arrangement structure shown in Figure 15 is used to demonstrate the movement of particles, changes in spatial position, and development of pores during the shearing process [53,54], and its accuracy has been confirmed by relevant research [41,55].

Consider two packing forms of uniform tailings, as shown in Figure 15a,b. Packing A represents dense tailings that dilate during the shearing process, and packing B represents loose tailings that contract during the shearing process. If the shearing process is induced under undrained conditions, packing A will have negative pore water pressure, and, at the same time, the mean effective stress will increase accordingly. Static liquefaction will not occur in packing A, while packing B will exhibit positive pore water pressure and may undergo static liquefaction upon decreasing the mean effective stress, as shown in Figure 14.

For tailings with a low FC (Figure 15c), the fines exist between the large particles. When shearing occurs, the fines will slip into the void in the skeleton formed by the large particles, which promotes the volume compression of the tailings, causing the pore water pressure to increase rapidly, and then static liquefaction occurs. If the FC is increased until the fines completely fill the void in the skeleton, tailings may experience volume expansion during the shearing process without static liquefaction (Figure 15d). In summary, compressibility plays a key role in determining whether static liquefaction of tailings can occur under undrained conditions.

Section 3.2 analyzes the dependence of compressibility on the initial state and fines content of the tailings. Figure 13b shows that compressibility increases with an increasing void ratio. This result can explain the decrease in η_IL_ with an increasing e_0_ shown in Figure 10b. However, η_IL_ decreases with an increasing p_0_′ (Figure 10a), which contradicts the increase in compressibility caused by increasing σ_v_′ (Figure 13b). In CUTC tests, the increment in pore water pressure can be obtained from Equation (10):

(11) du=dp−dp′

In the elastoplastic theory of soils [26],

(12) $$\left\{\begin{array}{c} {dp}^{\prime }=-d\varepsilon_{v}^{p}G\frac{2(1+\nu )}{3(1-2\nu )}\\ G=\frac{A}{e-e_{min}}{\left(\frac{p}{p_{ref}}\right)}^{b} \end{array}\right.$$

where $d\varepsilon_{v}^{p}$ is the plastic volumetric strain increment; G is the elastic shear modulus; ν is Poisson’s ratio; A, e_min_ and b are all material properties; and p_ref_ = 100 kPa according to convention. If the elastic deformation is neglected, then

(13) $$d\varepsilon_{v}^{p}=\frac{de}{1+e}$$

which is combined with Equation (8):

(14) $$d\varepsilon_{v}^{p}=\frac{md\sigma_{v}\prime }{1+e}$$

By analyzing Equations (11), (12), and (14), it can be concluded that the increase in pore water pressure is not only related to compressibility but also the elastic shear modulus. As the pressure grows, compressibility decreases, and the elastic shear modulus increases. Therefore, the magnitude of the increment in pore water pressure depends on the dominant factor. The tests results demonstrate that the elastic shear modulus was the dominant factor in this study.

According to Section 3.2, a threshold exists for the effect of FC on η_IL_. This contradicts the observation of continuously increasing compressibility with increasing FC (Figure 13c). Lade [49] studied Ottawa sand with fines and found that the compressibility causing static liquefaction varies with the FC, which certainly adds to the complexity of such studies.

The above analysis reveals that the compressibility defined in the OC tests poses limitations for analyzing static liquefaction. Firstly, no matter how dense the tailings material is, it will exhibit positive compressibility in the OC test. Therefore, compressibility cannot be used to determine whether the tailings will undergo shear dilation or contraction, nor can it determine whether the tailings will liquefy or remain stable. In other words, it lacks a boundary reference. Secondly, compressibility cannot comprehensively reflect the volume change trend of tailings, as the effect of shear stress is not considered. The change in pore water pressure during static liquefaction is mainly due to the change in the stress ratio, which is always kept constant in OC tests. Therefore, compressibility is helpful for understanding the static liquefaction phenomenon but not for the constitutive description and prediction of static liquefaction.

### 4.2. Relationship Between ψ and Static Liquefaction

Critical state soil mechanics provides a good framework for studying static liquefaction. The CSL, the final state of tailings under shear stress, provides a good boundary reference. The distance between the current state and the critical state can be used as a substitute for compressibility. ψ is the most commonly used descriptor for distance in this regard.

Figure 2 shows the CSL in the e-p space. The e-p space is divided into two regions by the CSL. The lower left region is the dense region with ψ < 0, and the upper right region is the loose region with ψ > 0. The larger the value of ψ, the looser the tailings. ψ = 0 indicates the critical state.

**Figure 16:**
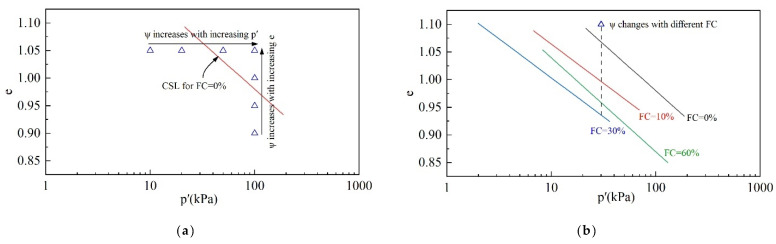
The variation in ψ with respect to the initial state and FC: (**a**) unique FC; (**b**) changing FC.

Figure 16a presents the relationship between ψ, e_0_, and p_0_′. The blue triangle represents the current state. At constant e_0_, as p_0_′ increases, the value of ψ increases. At constant p_0_′, ψ increases with the increasing e_0._

Figure 16b presents the relationship between ψ and FC. In a constant initial state, the value of ψ is related to the position of CSLs affected by FC. As FC increases, ψ increases first and then decreases.

**Figure 17:**
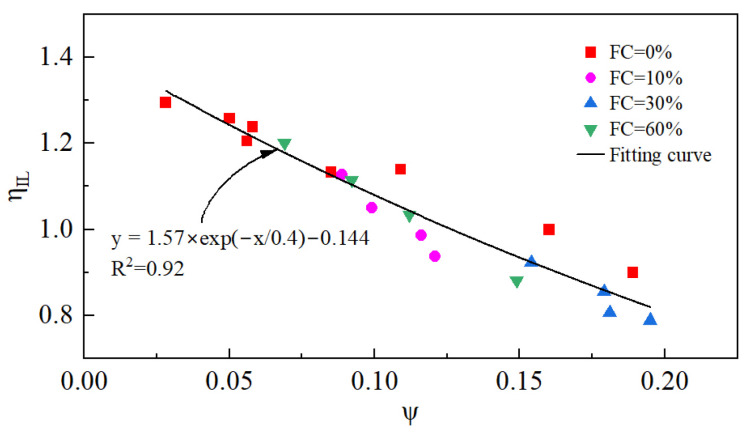
The variation in η_IL_ with respect to ψ.

The variation in η_IL_ with respect to e_0_, p_0_′, and FC in Section 3.1.3 can be well explained by ψ. Figure 17 shows the relationship between η_IL_ and ψ under various initial states and FCs. Obviously, η_IL_ is related to ψ, regardless of the initial state and FC. In summary, as ψ increases, the tailings become loose and η_IL_ decreases.

**Figure 18:**
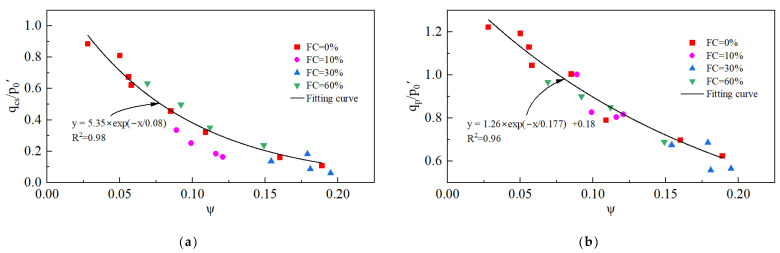
The variation in shear strengthen ratio with ψ: (**a**) critical state shear strength ratio; (**b**) undrained shear strength ratio.

The critical-state shear strength and undrained shear strength are strongly correlated with ψ, and the relationships are shown in Figure 18. As ψ increases, the tailings become loose, and the critical-state shear strength and undrained shear strength decrease accordingly.

**Figure 19:**
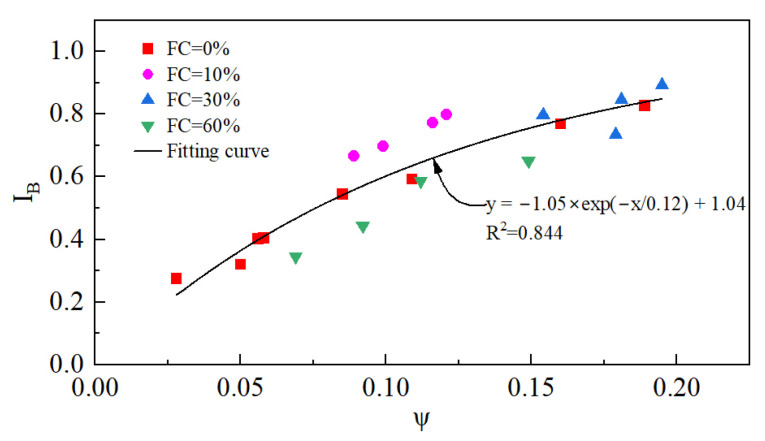
The relationship between I_B_ and ψ.

The relationship between the I_B_ and ψ is shown in Figure 19. It is clear that I_B_ increases with the increase in ψ, independent of the initial state and FC.

The following conclusions can be drawn from the above discussion wherein it is indicated that the combined effect of the initial state and the fines content of the tailings on static liquefaction can be normalized by the state parameter. As long as the state parameters of the tailings are known, the liquefaction behavior can be evaluated comprehensively, and this finding can be exploited to reduce the complexity of static liquefaction studies of tailings.

From the above research and discussion, it has been determined that the main reason for the static liquefaction of tailings is the increase in pore water pressure in the undrained condition. In order to effectively prevent the static liquefaction of tailings, it is necessary to strictly control the drainage conditions of tailings and detect the pore pressure of tailings to prevent an excessive rise in pore water pressure.

## 5. Conclusions

In this study, the effects of the initial state and fines content on the static liquefaction of tailings were investigated using CUTC tests and OC tests. The main conclusions are as follows.

(1) Saturated loose tailings can undergo static liquefaction in undrained conditions. The instability line, undrained shear strength, critical state strength, and brittleness index are associated with the initial state and the fines content. A larger void ratio and higher mean effective stresses are more likely to initiate static liquefaction. There exists a fines content that is most likely to initiate static liquefaction, which is 30% in this study.
(2) The instability line slope decreases with an increasing void ratio and mean effective stress. The fines content has a threshold, and the instability line slope decreases and then increases with an increasing fines content and reaches a minimum at an FC = 30%. The brittleness index has an opposite variation pattern to the instability line slope and reaches a peak at an FC = 30%.
(3) The fines content has no effect on the critical state stress ratio. In the e-lnp′ space, the critical-state lines at different fines contents are approximately parallel, and the position of the critical-state line drops and then rises with an increasing fines content.
(4) The compressibility of the tailings increases with an increasing void ratio and fines content and decreases with increasing pressure. Compressibility poses limitations in evaluating the static liquefaction of tailings and has little contribution to the constitutive description and prediction of static liquefaction.
(5) The state parameter is an effective indicator for evaluating the static liquefaction of tailings containing fines. The comprehensive influence of the initial state and fines content on static liquefaction can be normalized by the state parameter.

These findings have guiding significance for the design and monitoring of and development of risk warnings for tailings dams, providing an experimental and theoretical basis for the stability assessment of existing tailings dams. We must acknowledge that there are certain limitations to our research in this article, which prompts us to focus on the next research direction, namely, how the particle grading and shape of coarse particles affect the static liquefaction of tailings, and to integrate discrete element simulations to identify the mechanical mechanisms involved.

## Figures and Tables

**Table 1:** Basic physical properties of the tailings.

Sampling Point	Particle Mass Percentage (%)			Median Size d_50_ (mm)	Particle Specific Gravity	Minimum Dry Density (g/cm^3^)	Maximum Dry Density (g/cm^3^)
	<0.005 mm	0.005 mm–0.075 mm	>0.075 mm				
P1	3.56	68.97	27.47	0.039	2.79	1.158	1.567
P2	4.33	70.29	25.38	0.038	2.79	1.143	1.548
P3	5.05	71.72	23.23	0.037	2.80	1.127	1.527
P4	5.86	73.03	21.11	0.036	2.80	1.111	1.502
P5	6.69	74.36	18.95	0.034	2.80	1.094	1.469

**Table 2:** Summary of CUTC tests.

FC (%)	e_0_	p_0_′ (kPa)	ψ_0_
0	1.089	50	0.058
	1.085	100	0.109
	1.090	200	0.16
	1.087	300	0.189
	0.958	300	0.085
	0.928	300	0.028
	1.036	100	0.056
	0.979	200	0.05
10	1.088	50	0.121
	1.041	100	0.116
	0.981	200	0.099
	0.947	300	0.089
30	1.101	50	0.195
	1.044	100	0.181
	0.975	200	0.154
	0.923	300	0.179
60	1.071	50	0.149
	0.978	100	0.112
	0.901	200	0.092
	0.846	300	0.069
80	1.085	50	−0.102
	0.979	100	−0.12
	0.898	200	−0.109
	0.855	300	−0.099

e_0_, p_0_′, and ψ_0_ are post-consolidation void ratio, mean effective stress, and state parameter, respectively.

**Table 3:** Summary of OC tests.

FC (%)	e_i_			
0	1.128	1.050	0.879	0.799
10	1.128	0.945	0.830	0.691
30	1.128	0.915	0.828	0.717
60	1.128	1.076	0.940	0.837
80	1.128	1.228	1.050	0.952

e_i_ is initial void ratio.

## Data Availability

The datasets presented in this article are not readily available because the data are part of an ongoing study.
